# Laser-Sintered Mg-Zn Supersaturated Solid Solution with High Corrosion Resistance

**DOI:** 10.3390/mi12111368

**Published:** 2021-11-06

**Authors:** Youwen Yang, Wei Wang, Mingli Yang, Yingxin Yang, Dongsheng Wang, Zhigang Liu, Cijun Shuai

**Affiliations:** 1 Institute of Bioadditive Manufacturing, Jiangxi University of Science and Technology, Nanchang 330013, China; yangyouwen@jxust.edu.cn (Y.Y.); wangwei@mail.jxust.edu.cn (W.W.); yangmingli@mail.jxust.edu.cn (M.Y.); 9519930003@jxust.edu.cn (Y.Y.); 2 Key Laboratory of Construction Hydraulic Robots of Anhui Higher Education Institutes, Tongling Univesity, Tongling 244061, China; wangdongsheng@tlu.edu.cn; 3 School of Electrical Engineering and Automation, Jiangxi University of Science and Technology, Ganzhou 341000, China; 4 State Key Laboratory of High Performance Complex Manufacturing, Central South University, Changsha 410083, China; 5 Double Medical Technology Inc., Xiamen 361026, China

**Keywords:** Mg-Zn solid solution, laser sintering, mechanical alloying, corrosion resistance

## Abstract

Solid solutions of Zn as an alloy element in Mg matrixes are expected to show improved corrosion resistance due to the electrode potential being positively shifted. In this study, a supersaturated solid solution of Mg-Zn alloy was achieved using mechanical alloying (MA) combined with laser sintering. In detail, supersaturated solid solution Mg-Zn powders were firstly prepared using MA, as it was able to break through the limit of phase diagram under the action of forced mechanical impact. Then, the alloyed Mg-Zn powders were shaped into parts using laser sintering, during which the limited liquid phase and short cooling time maintained the supersaturated solid solution. The Mg-Zn alloy derived from the as-milled powders for 30 h presented enhanced corrosion potential and consequently a reduced corrosion rate of 0.54 mm/year. Cell toxicity tests confirmed that the Mg-Zn solid solution possessed good cytocompatibility for potential clinical applications. This study offers a new strategy for fabricating Mg-Zn solid solutions using laser sintering with MA.

## 1. Introduction

Mg-based alloys are recognized as revolutionary biomaterials because of their natural degradability, high specific strength, suitable Young’s modulus, and favorable biocompatibility [1,2,3]. In order to increase their clinical applicability, one issue deserving greater attention is their rapid degradation in vivo [4,5]. In fact, the rapid degradation of Mg is mainly due to its low electrode potential [6,7]. According to electrochemical kinetics, a solid solution of high electrode potential substances (such as Zn, Fe) in a Mg matrix is able to improve the overall electrode potential, thereby enhancing the corrosion resistance and reducing the degradation [8,9]. Zn is a biometal that fulfills a series of vital biofunctions in vivo [10,11]. It has been reported that Zn is able to stimulate new bone growth [12]. Nevertheless, the solubility of alloy elements, including Zn in Mg, is very limited (less than 1 wt.% at room temperature) [13]. As such, direct alloying with Zn will cause precipitation of the intermetallic phase and adverse formation of galvanic corrosion, thereby accelerating the degradation.

In order to enhance the corrosion potential of Mg, it is necessary to increase the solid solubility of Zn in the Mg matrix. Mechanical alloying (MA) is a non-equilibrium powder solid-state alloying method, which can improve the solid solubility of the second component in the parent phase to form a supersaturated solid solution [14]. During MA, the powders form composite particles with a layered structure through continuous deformation, fragmentation, and welding [15,16]. In this condition, the lamellar spacing is decreased, reducing the diffusion distance of solid atoms, thereby speeding up the alloying process [17]. At the same time, the mechanical force causes massive strains and defects in the particles and increases the chemical activity of the powders. As a consequence, the energy barrier of atomic diffusion is reduced. In a previous study, a channel was further provided for the rapid diffusion of alloying elements [18].

In fact, MA-prepared powders should be consolidated into samples with desirable shapes to achieve specific functions. Laser sintering, as a laser-additive manufacturing process, is able to fabricate parts with complicated structures [19,20,21]. It utilizes laser energy to partially melt the powder particles to obtain a limited liquid molten pool, which subsequently undergoes fast cooling. It is expected that the partial melting mechanism and rapid solidification are able to maintain the original supersaturated solid solution, thereby obtaining Mg-Zn parts with high corrosion resistance [22].

According to the above considerations, in this study, Mg-Zn powders were firstly prepared using MA, which were subsequently shaped into samples using laser sintering. The microstructures of the MA-processed Mg-Zn supersaturated solid solution powders were investigated. The corrosion behavior of the laser-sintered Mg-Zn parts was investigated using electrochemical tests and immersion experiments. Additionally, in vitro cell tests were carried out to assess the biocompatibility of the parts for potential bone implant applications.

## 2. Materials and Methods

### 2.1. MA Processing of Mg-Zn Powder

In this study, spherical Mg powders (15–53 μm, 99%) and Zn powders (~10 μm, 99%) supplied from Tangshan Wei Hao Co., Ltd., in China were used as the original materials. The Mg-Zn mixture powders with 14 wt.% Zn were synthesized using a planetary ball mill from Retsch GmbH (Dusseldorf, Germany). The ratio of the ball to powder weight was 10:1 and the vial speed was 350 rpm. During MA, 3 wt.% of alcohol was added to the vial to avoid the occurrence of cold welding. Additionally, the milling process was interrupted for 5 min out of every 20 min to avoid overheating.

The surfaces of as-milled Mg-Zn powders after 10, 20, 30 and 40 h were observed via scanning electron microscope (SEM, EVO 18) equipped with an energy-dispersive spectrometer (EDS, FEI Nova Nano SEM450) at 20 kV. The cross-section of the as-prepared powders was studied using SEM. In detail, the Mg-Zn powders were inlaid using a hot inlay machine and polished to 2000 grit using metallographic sandpaper. Furthermore, the microstructure of Mg-Zn powders was analyzed via transmission electron microscope (TEM, JEM2100, Tokyo, Japan) at 200 kV. The phase composition was researched utilizing a D8 Advance X-ray diffractometer (XRD) with Cu-Kα radiation. The scanning speed and scanning range were set to 5°/min and 20~80°, respectively.

### 2.2. Laser Processing of Mg-Zn Samples

**Figure 1:**
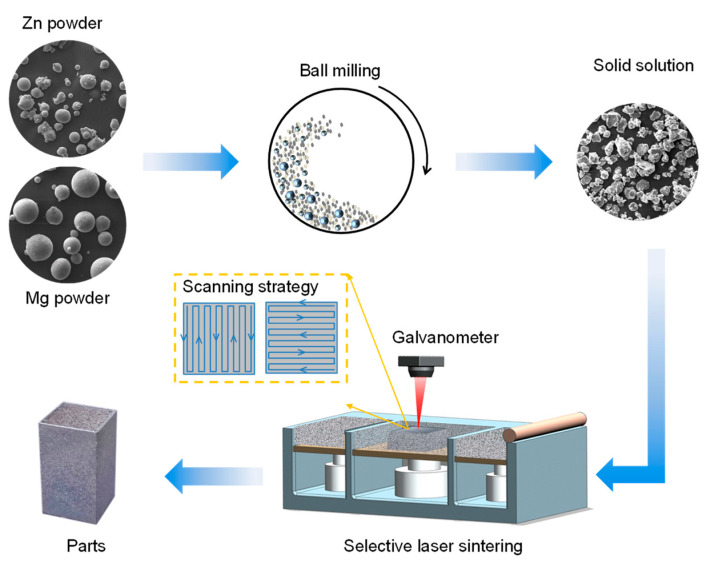
Schematic map showing the preparation process for laser-sintered Mg-Zn parts.

The as-prepared powders after milling for 10, 20, and 30 h were adopted for laser experiments, as schematically shown in Figure 1. The system was composed of a fiber laser, a closed glove box, and a computer control system. The laser adopted in this work was a YLR-500-WC (IPG, Burbach, Germany), which is a continuous wave laser. The spot size was about 60 μm. After a series of pilot experiments, the cubic parts were fabricated at a laser power of 62 W, a scanning rate of 110 mm/s, a hatching space of 50 μm, and a layer thickness of 50 μm. During laser processing, high purity argon was offered at a flow rate of 0.1 L/min to mitigate the oxidation. To facilitate the description, the as-built Mg-Zn parts that developed from as-milled powders for 0, 10, 20, and 30 h were designated as M0, M10, M20, and M30, respectively.

### 2.3. Electrochemical Tests

The laser-sintered samples were polished to 2000 grit with metallographic sandpaper and then cleaned with ultrasonic waves before drying. The electrochemical testing was carried out in simulated body fluid (SBF) using a three-electrode electrochemical workstation (CHI660C, Chenhua Instruments Inc., Shanghai, China). The testing samples, platinum sheet, and Ag/AgCl acted as the working electrode, counter electrode, and reference electrode, respectively. The samples were treated at open circuit potential for 5 min to obtain stable open potential. Then, the Tafel polarization curve was obtained by polarization test at a rate of 1 mV/s. The corrosion current density and potential were analyzed by fitting the Tafel polarization curve using NOVA software. Electrochemical impedance spectroscopy (EIS) was measured in the range of 100 kHz to 0.01 Hz, and the corresponding Nyquist and Bode diagrams were obtained.

### 2.4. Immersion Experiments

The immersion experiment was carried out in SBF at 37 °C. The ratio of the solution volume to the exposed area was 20 mL/cm^2^. During immersion, the pH of the immersion solution was measured using a pH meter every 24 h. The concentrations of Mg^2+^ and Zn^2+^ in solution were detected using an inductively coupled plasma optical emission spectrometer (ICP-OES, Cambridge, UK). After immersion for 7 d, the corrosion surface was observed using SEM and the composition of the corrosion product was analyzed using EDS. Then, the corrosion products were removed using chromic acid consisting of 200 g/L of CrO_3_ and 10 g/L of AgNO_3_. The corrosion rate (R_corr_) was calculated via the weight loss method [23]:

(1) $$R_{\mathrm{corr}}=\frac{8.76\times 104\;w}{s\rho t},$$

where w, t, and s are weight loss, the immersion time, and the area exposed to the solution, respectively. Here, ρ represents the density of the specimens.

### 2.5. Toxicity Tests

MG-63 cells were used to evaluate the cytocompatibility. The samples were immersed into DMEM medium containing 10% fetal bovine serum, 1% penicillin, and streptomycin. The ratio of the exposure area to solution was 1.25 cm^2^/mL and the extract was obtained after immersion for 3 days. Then MG-63 cells were cultured in the extracts at 37 °C and 5% CO_2_ for 1, 3, and 5 d, respectively. Afterwards, the cells were stained with Calcein-AM/PI for 30 min and then observed using a fluorescence microscope (Olympus Co., Ltd., Tokyo, Japan). In addition, the cell viability was evaluated using a cell counting kit-8 (CCK-8) assay. Cells were cultured in 24-well culture plates containing DMEM for 1 day and replaced with extracts to further culture them for 1, 3, and 5 d, respectively. After this, the CCK-8 solution was dropped into each well for 3 h and the absorbance at 450 nm was measured using a microplate reader (Beckman, Brea, CA, USA).

### 2.6. Statistical Analysis

All tests were performed 3 times to achieve the averages and the obtained data were displayed as means ± standard deviation. Statistical significance was estimated using SPSS 20.0 soft, where *p* < 0.05 was considered to be of statistical difference.

## 3. Results

### 3.1. Microstructure of Mg-Zn Mixed Powders

**Figure 2:**
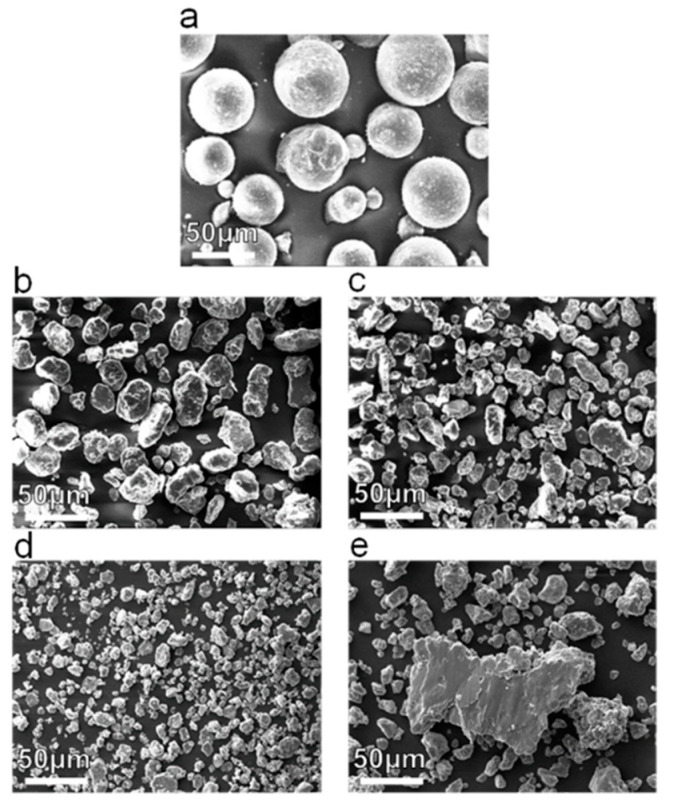
SEM morphologies of (**a**) initial mixed powders and (**b**–**e**) mixed powders after milling for 10, 20, 30, and 40 h, respectively.

The morphologies of Mg-Zn mixed powders before and after milling are shown in Figure 2. The initial mixed powders exhibited a regular spherical shape, as shown in Figure 2a. After milling for 10 h, the powders obviously changed into a flat block shape owing to the impact of the powerful mechanical forces, as presented in Figure 2b. As the milling time increased to 20 and 30 h, the powders fractured because of the continuous collision and deformation, thereby reducing the particle size and generating numerous new particle surfaces, as shown in Figure 2c,d. In particular, some particles were welded together, considerably reducing the inter-layer space, which was favorable for the solution atom diffusion. However, as the milling time further increased to 40 h, the as-milled powders experienced severe cold welding, as confirmed by the enlarged particle size shown in Figure 2e. Usually, the fracturing and welding process would achieve a balance over excessive milling periods, resulting in work hardening, which should be avoided during MA.

**Figure 3:**
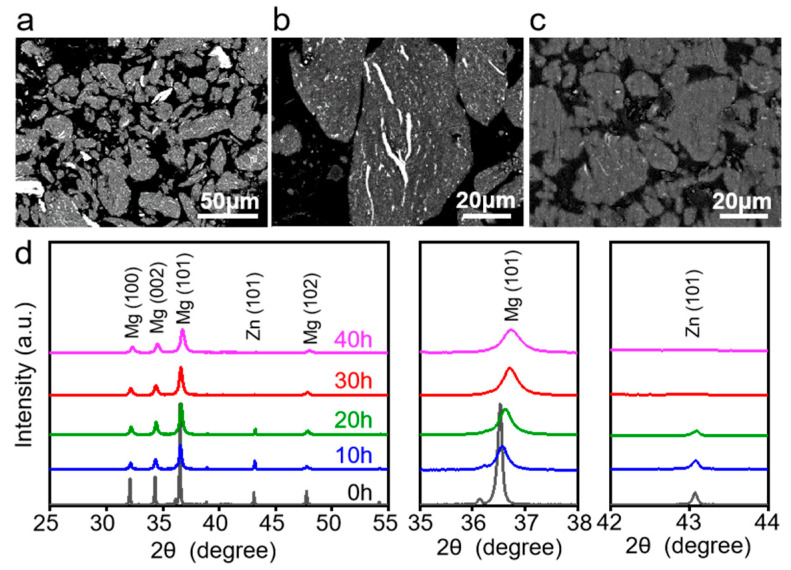
SEM image showing the cross-sections of Mg-Zn powders after milling for (**a**) 10 h, (**b**) 20 h, and (**c**) 30 h. (**d**) XRD patterns of as-milled powders.

The cross-section morphologies of Mg-Zn powders were investigated via SEM in back scattered mode. Herein, the particles were distinguished by dark contrast areas for Mg particles and bright contrast areas for Zn particles. Clearly, the majority of particles remained unalloyed after a short milling time of 10 h, as shown in Figure 3a, since the mechanical collisions and kinetic energy input were inadequate. After milling for 20 h, some Zn particles were trapped in Mg particles, as shown in Figure 3b, whereby the Zn particles showed a fine and lamellar-like structure, indicating severe deformation under the sustained collision of the grinding balls. As the milling time further increased to 30 h, the Zn particles almost disappeared. It was indicated that Zn particles were completely dissolved in the Mg matrix, forming a homogeneous distribution.

The phase compositions of the as-milled Mg-Zn powders were investigated, with the collected XRD spectrum exhibited in Figure 3d. Strong Mg and Zn diffraction peaks were detected after milling for 10 h. With the increase in milling time, the Zn diffraction peaks clearly became weaker, since partial Zn particles were gradually dissolved into the Mg matrix with the aid of continuous mechanical energy input [24]. A careful examination of the XRD spectrum showed that the primary diffraction peak of Mg shifted towards a higher 2θ position during MA. It was believed that the Zn atoms were dissolved in the Mg matrix, which caused lattice distortion [25]. Notably, the Zn diffraction peaks disappeared after milling for 30 h, which further confirmed the formation of a homogeneous Mg-Zn solid solution.

Based on previous XRD data, the lattice strain, crystallite size, and lattice parameters for as-milled Mg-Zn powders were calculated utilizing the Scherer formula and Williamson–Hall formula, as follows [26]:

(2) $$T=\frac{0.89\cdot \lambda }{b\cdot \cos\delta }$$

(3) $$b\cdot \cos\theta =\frac{K\cdot \lambda }{T}+4\cdot \varepsilon \cdot \sin\delta$$

where *T* is the crystallites size, *b* is half maximum width, *δ* is the peak angle, *ε* is the lattice strain, and *K* and *λ* are constants. As listed in Table 1, as the milling time gradually increased, the lattice strain increased from 7.43 × 10^−4^ to 23.15 × 10^−4^. Meanwhile, the crystallite size of the Mg was gradually refined to 24.2 nm at 30 h, although no significant variation occurred at 40 h, owing to the predominance of cold welding at this stage. Additionally, the lattice parameters (a, c) of Mg declined at the initial 30 h point and similarly remained unchanged at 40 h. These results prove that MA caused numerous lattice distortion or defects, which was beneficial in promoting the diffusion of Zn atoms into the Mg matrix, thereby forming the Mg-Zn supersaturated solid solution.

**Figure 4:**
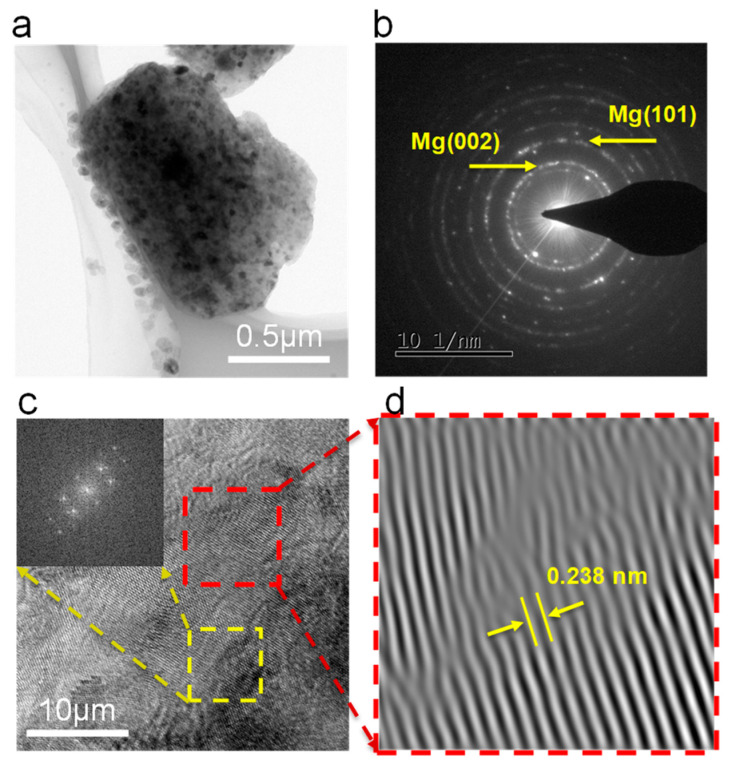
TEM analysis of Mg-Zn powder after milling for 30 h: (**a**) bright-field TEM image; (**b**) SAED pattern; (**c**) high-resolution image with FFT inset; (**d**) corresponding inverse FFT image.

The microstructure of the Mg-Zn powder after milling for 30 h was studied utilizing TEM, as shown in Figure 4. The powder exhibited a nano-scaled grain size, as shown in Figure 4a. The Debye–Scherrer rings are shown in Figure 4b, proving the existence of (101)_Mg_ and (002)_Mg_. The HRTEM image with the fast Fourier transformation (FFT) inset is presented in Figure 4c. Obviously, some lattice distortions were observed in the red region, while the interplanar spacing was measured for 0.238 nm. This value was smaller than for pure Mg (0.2452 nm) [27]. Additionally, the FFT image of the yellow area shows a hexagonal close-packed structure, suggesting good structural integrity of the magnesium matrix. The majority of the lattice dislocations were caused by the solid solution of Zn. These results directly confirm the formation of the Mg-Zn supersaturated solid solution after MA.

### 3.2. Microstructure of As-Build Samples

**Figure 5:**
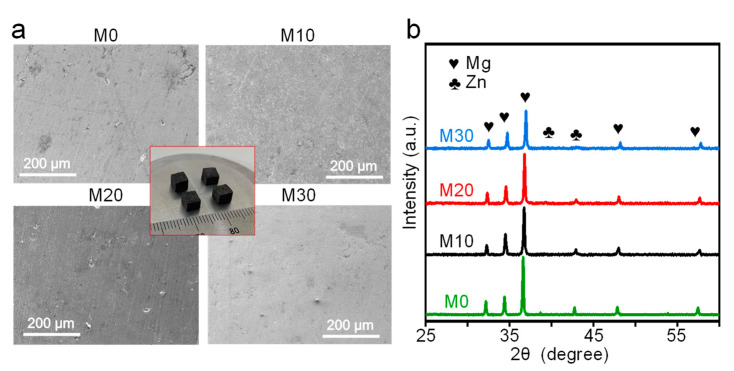
(**a**) Laser-sintered parts and corresponding surfaces after polishing. (**b**) XRD patterns of laser-sintered samples.

Laser-sintered samples were fabricated using as-milled powders and the corresponding surfaces after polishing are presented in Figure 5a. It can be observed that there were some microspores in the parts, since the particles were partially melted. Nevertheless, a relatively more homogeneous microstructure was observed for M30, since the as-milled powder at 30 h was refined and was beneficial for the laser forming. The phase composition was investigated using XRD, as shown in Figure 5b. It was found that the XRD patterns of the sintered parts were basically similar to those of the as-milled powders. Particularly, no diffraction peak corresponding to the Zn phase was detected in the M30 part, which indicated that the Mg-Zn solid solution still existed after laser shaping. The laser sintering involved a partial melting mechanism, in which the as-milled powders were partially melted. Subsequently, the molten pool with the limited liquid phase experienced a rapid solidification, which was able to limit the precipitation of the solid solution [28]. As such, the Mg-Zn solid solution was maintained after laser processing.

### 3.3. Electrochemical Behavior

**Figure 6:**
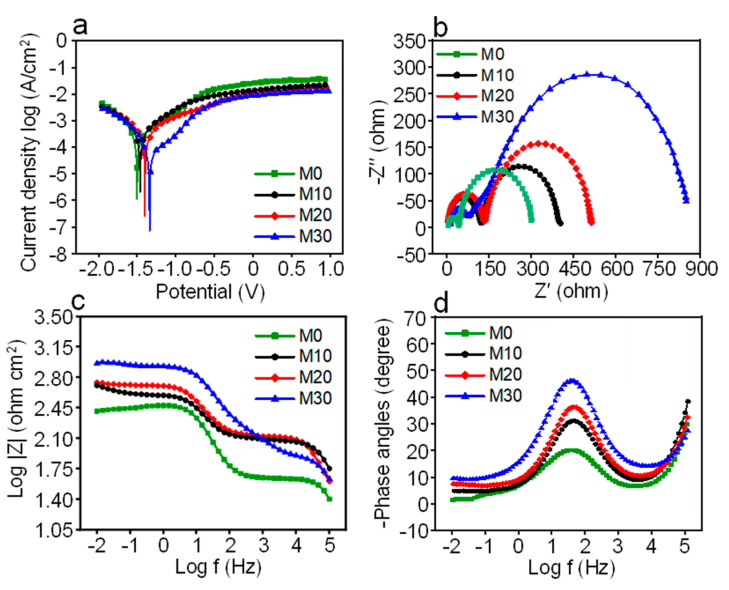
(**a**) Potentiodynamic polarization curves. (**b**) Nyquist diagrams. (**c**) Bode impedance plots. (**d**) Bode phase angle curves.

The corrosion behavior of the sintered parts was investigated via electrochemical experiments in SBF solution. The polarization curves for all parts are displayed in Figure 6a, and the corresponding corrosion potential (E_corr_) and corrosion current density (I_corr_) were calculated via Tafel region extrapolation, as listed in Table 2. The M0 specimen possessed a relatively low E_corr_ of −1.50 ± 0.06 V. Nevertheless, the E_corr_ corresponding to M10, M20, and M30 shifted positively and reached a maximum of −1.34 ± 0.02 V for M30, which revealed a relatively low corrosion tendency. The Mg matrix with the maximum atomic density originated from the solid solution of Zn atoms, which led to the crystallographic texture change, thereby reducing its surface energy [29]. In this case, the low surface energy required massive dissolution activation energy to cause matrix degradation, which had an effective protective effect [30]. In contrast, the I_corr_ evidently decreased to 16.4 ± 1.3 μA/cm^2^ as compared with M0 (61.7 ± 2.8 μA/cm^2^), indicating superior corrosion resistance.

An electrochemical impedance spectroscopy (EIS) test was performed, with the obtained results presented in Figure 6b–d. All Nyquist curves presented two typical impedance loops across the whole frequency range, revealing the similar degradation mechanisms. No obvious differences were exhibited over the high-frequency region for the as-built samples, indicating the same dissolution process, as shown in Figure 6b. However, the M0 sample possessed a relatively small capacitive arc in the low-frequency region, which indicated a low charge transfer resistance [31]. In contrast, the diameter of the capacitive arcs corresponding to M10, M20, and M30 gradually increased to the maximum point. As evident in Figure 6c, a relatively large impedance moduli appeared in the low-frequency region. Furthermore, the phase angle values in the low-frequency region also reached the maximum point, as shown in Figure 6d, which revealed an improvement of the charge transfer resistance for M30 [31]. Notably, the phase angle values in the high-frequency region were similar and far below 60° for all samples, indicating the formation of porous oxide film during the corrosion process [32].

### 3.4. Degradation Performance

**Figure 7:**
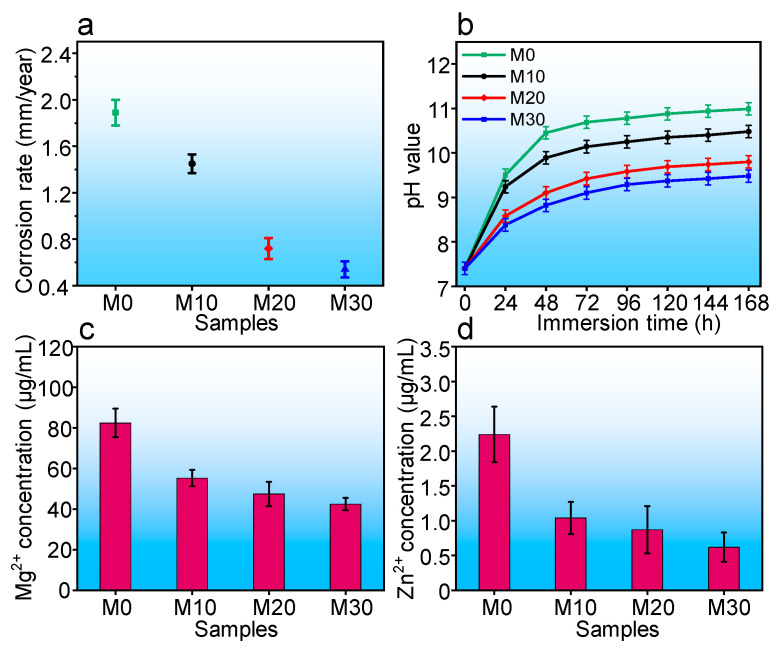
(**a**) Corrosion rates, (**b**) pH values, and (**c**) Mg^2+^ and (**d**) Zn^2+^ ion concentrations for all sample extracts.

To evaluate the degradation behavior, the immersion tests were employed in SBF solution. The corrosion rates were calculated, with the results displayed in Figure 7a. Clearly, the corrosion rates corresponding to M10, M20, and M30 gradually decreased to 0.54 mm/year as compared with M0 at 1.89 mm/year. The pH values of SBF solution at different immersion time were collected, as shown in Figure 7b. The pH the M0 group initially increased rapidly, then increased slowly and finally stabilized at 10.9. In contrast, the pH levels corresponding to M10, M20, and M30 increased slowly and stabilized at 10.3, 9.7, and 9.3, respectively. The results were ascribed to the generation of brucite and Mg-containing calcium phosphate, which precipitated on the Mg matrix surface, hindering the matrix corrosion [33,34,35,36]. Furthermore, the Mg^2+^ and Zn^2+^ ion concentrations after immersion for 168 h are exhibited in Figure 7c. The Mg^2+^ and Zn^2+^ ion concentrations of M10, M20, and M30 groups were evidently lower than the M0 group. Especially for the M30 group, both the Mg^2+^ and Zn^2+^ ion concentrations reached 42.5 and 0.62 μg/mL, respectively, which indicated reduced degradation rates.

**Figure 8:**
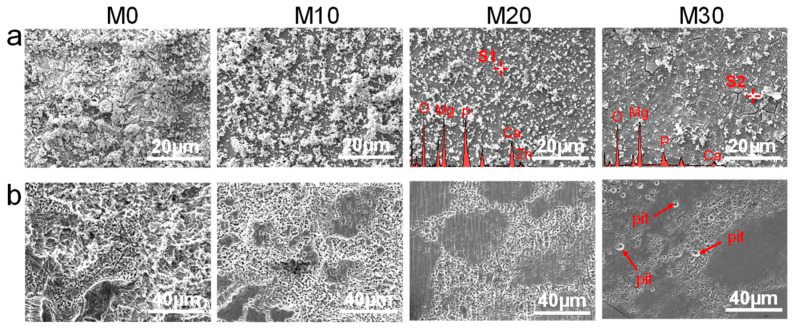
Surface morphologies of laser-sintered parts: (**a**) before and (**b**) after removing corrosion products.

The typical corrosion surfaces for all samples after immersion for 7 d are exhibited in Figure 8a. It can be seen that a large amount of corrosion products were deposited on the M0 matrix, indicating intense corrosion. The corrosion products obviously decreased for M10, M20, and M30. Only a small amount of agglomerations appeared on the surface of M20 and M30, which was attributed to the change of the surface chemical composition. Analyzing the EDS results detected for area S1 in M20 and area S2 in M30, the agglomerations mainly included large amounts of O, Mg, P, and Ca, as well as small amount of Zn for M20, while almost no additional Zn was detected for M30. Furthermore, massive continuous and wide cracks existed in the corrosion films grown on M20. In contrast, discontinuous and narrow cracks were observed for M30, which was affected by the dehydrogenation process.

To further analyze the degradation mechanism, the surface morphologies after removal of corrosion products are displayed in Figure 8b. Obviously, the precipitated phase remained unattacked while the adjacent Mg matrix seriously dissolved from the matrix surface to the center, which led to numerous deep pits for the M0 sample. Some large cavities were even formed owing to the strong reactions of galvanic corrosion. The precipitated phase increased the potential difference of the corrosion couple with the Mg matrix, thereby accelerating the degradation [37,38,39]. Comparatively, the corrosion degree corresponding to M10, M20, and M30 samples gradually decreased. For M30, only a few shallow and small corrosion pits were present on the matrix surface, which indicated significantly enhanced corrosion resistance and a uniform degradation mode.

### 3.5. Cytocompatibility

**Figure 9:**
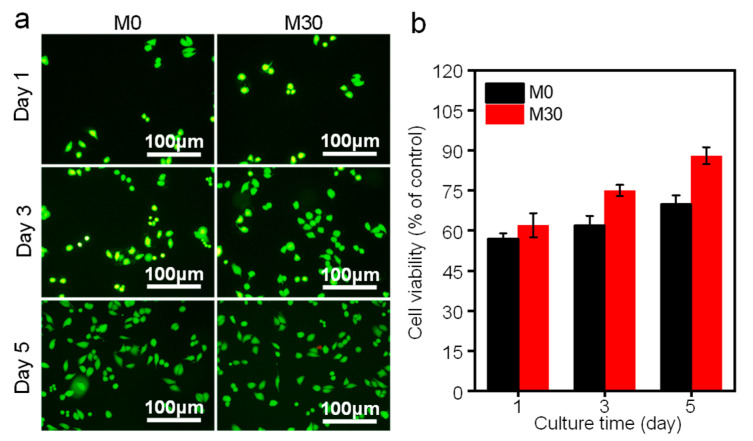
(**a**) Cell morphology and (**b**) cell viability results for MG-63 cells after 1, 3, and 5 d culture in M0 and M30 extracts.

To evaluate the proliferation of MG-63 cells cultured in the extracts of M0 and M30, cells were stained after culturing for 1, 3, and 5 d. The fluorescence image in Figure 9a shows live cells as green and dead cells as red, respectively. It can be seen that the cell densities for both M0 and M30 groups increased with increasing culture time, indicating the favorable biocompatibility of the Mg-Zn alloy, despite the morphologies of MG-63 cells showing round and unhealthy shapes in M0 and M30 after culturing for 1 d. However, some spindle-shaped cells with filopodia were observed after 5 d. Notably, there were more live cells in the M30 group than in the M0 group, implying better cytocompatibility of M30. CCK-8 assay was carried out and the results are shown in Figure 9b. Overall, the viability of M30 was higher than that of M0. In detail, the cell viability levels for M0 were 57% at day 1, 62% at day 3, and 70% at day 5, respectively, indicating relatively low cell viability. This was attributed to the fast degradation rate leading to increased pH, which was harmful to cell growth [40,41,42]. Regarding M30, the cell viability reached 62% at day 1, 75% at day 3, and 88% at day 5. It could be concluded that M30 showed better cytocompatibility for cell growth in comparison to M0. The reason was the improvement of the solid solubility of Zn in Mg, which contributed to better corrosion resistance, resulting in a mild environment for cell growth.

## 4. Conclusions

In the present study, Mg-Zn solid solution powders were prepared using MA. During MA, the continuous and mandatory mechanical grinding introduced numerous lattice defects and the diffusion channel of Zn into the Mg matrix, resulting in supersaturated solid solution powders after milling for 30 h. The as-prepared powders were then developed into parts using laser sintering, which was a typical feature of the Mg-Zn supersaturated solid solution due to the fast cooling rate. The results showed that the laser-sintered Mg-Zn parts presented relatively high corrosion potential and anti-charge transfer ability. The corrosion rate was improved to 0.54 mm/year. The parts also showed good cytocompatibility.

## Figures and Tables

**Table 1:** Crystallite size, lattice strain, and constant values of the Mg matrix.

Milling Time	Lattice Strain	Crystallite Size (nm)	Lattice Parameters (Å)	
			a	c
0 h	7.43 × 10^−4^	68.2	3.2049	5.2105
10 h	16.60 × 10^−4^	37.9	3.2032	5.2001
20 h	20.56 × 10^−4^	26.8	3.2001	5.1952
30 h	23.31 × 10^−4^	24.2	3.1973	5.1898
40 h	23.15 × 10^−4^	24.6	3.1971	5.1892

**Table 2:** Tafel fitting results derived from the polarization curves of laser-sintered specimens.

Samples	E_corr_ (V)	I_corr_ (μA/cm^2^)
M0	−1.50 ± 0.06	61.7 ± 2.8
M10	−1.46 ± 0.03	−1.46 ± 0.03
M20	−1.40 ± 0.04	31.4 ± 2.5
M20	−1.34 ± 0.02	16.4 ± 1.3

## Data Availability

The data presented in this study are available on request from the corresponding author.

## References

[1] Xu L., Zhang E., Yin D., Zeng S., Yang K. (2008). In vitro corrosion behaviour of Mg alloys in a phosphate buffered solution for bone implant application. J. Mater. Sci. Mater. Med..

[2] Yu Y., Lu H., Sun J. (2018). Long-term in vivo evolution of high-purity Mg screw degradation—Local and systemic effects of Mg degradation products. Acta Biomater..

[3] He R., Liu R., Chen Q., Zhang H., Wang J., Guo S. (2018). In vitro degradation behavior and cytocompatibility of Mg-6Zn-Mn alloy. Mater. Lett..

[4] Zeng R.-C., Li X.-T., Li S.-Q., Zhang F., Han E.-H. (2015). In vitro degradation of pure Mg in response to glucose. Sci. Rep..

[5] Myrissa A., Agha N.A., Lu Y., Martinelli E., Eichler J., Szakács G., Kleinhans C., Willumeit-Römer R., Schäfer U., Weinberg A.-M. (2016). In vitro and in vivo comparison of binary Mg alloys and pure Mg. Mater. Sci. Eng. C.

[6] Li H., Wen J., He J., Shi H., Liu Y. (2020). Effects of Dy Addition on the Mechanical and Degradation Properties of Mg–2Zn–0.5 Zr Alloy. Adv. Eng. Mater..

[7] Feng P., Kong Y., Liu M., Peng S., Shuai C. (2021). Dispersion strategies for low-dimensional nanomaterials and their application in biopolymer implants. Mater. Today Nano.

[8] Shuai C., He C., Qian G., Min A., Deng Y., Yang W., Zang X. (2021). Mechanically driving supersaturated Fe–Mg solid solution for bone implant: Preparation, solubility and degradation. Compos. Part Eng..

[9] Yang Y., Lu C., Peng S., Shen L., Wang D., Qi F., Shuai C. (2020). Laser additive manufacturing of Mg-based composite with improved degradation behaviour. Virtual Phys. Prototyp..

[10] Li H., Peng Q., Li X., Li K., Han Z., Fang D. (2014). Design, Microstructures, mechanical and cytocompatibility of degradable Mg–Zn based orthopedic biomaterials. Mater. Des..

[11] Qin H., Zhao Y., An Z., Cheng M., Wang Q., Cheng T., Wang Q., Wang J., Jiang Y., Zhang X. (2015). Enhanced antibacterial properties, biocompatibility, and corrosion resistance of degradable Mg-Nd-Zn-Zr alloy. Biomaterials.

[12] Qiao Y., Zhang W., Tian P., Meng F., Zhu H., Jiang X., Liu X., Chu P. (2014). Stimulation of bone growth following zinc incorporation into biomaterials. Biomaterials.

[13] Kubasek J., Vojtech D. (2013). Structural characteristics and corrosion behavior of biodegradable Mg-Zn, Mg-Zn-Gd alloys. J. Mater. Sci. Mater. Med..

[14] Suryanarayana C. (2018). Phase formation under non-equilibrium processing conditions: Rapid solidification processing and mechanical alloying. J. Mater. Sci..

[15] Varalakshmi S., Kamaraj M., Murty B.S. (2010). Processing and properties of nanocrystalline CuNiCoZnAlTi high entropy alloys by mechanical alloying. Mater. Sci. Eng..

[16] Othman A.R., Sardarinejad A., Masrom A.K. (2015). Masrom, Effect of milling parameters on mechanical alloying of aluminum powders. Int. J. Adv. Manuf. Technol..

[17] Amram D., Schuh C.A. (2020). Mechanical alloying produces grain boundary segregation in Fe–Mg powders. Scr. Mater..

[18] Lu L., Lai M.O., Zhang S. (1997). Diffusion in mechanical alloying. J. Mater. Process. Technol..

[19] Yap C.Y., Chua C.K., Dong Z.L., Liu Z.H., Zhang D.Q., Loh L.E., Sing S.L. (2015). Review of selective laser melting: Materials and applications. Appl. Phys. Rev..

[20] Bremen S., Meiners W., Diatlov A. (2012). Selective laser melting: A manufacturing technology for the future?. Laser Tech. J..

[21] Wang D., Ye G., Dou W., Zhang M., Yang Y., Mai S., Liu Y. (2020). Influence of spatter particles contamination on densification behavior and tensile properties of CoCrW manufactured by selective laser melting. Opt. Laser Tech..

[22] Sun S., Liu P., Hu J., Hong C., Qiao X., Liu S., Zhang R., Wu C. (2019). Effect of solid solution plus double aging on microstructural characterization of 7075 Al alloys fabricated by selective laser melting (SLM). Opt. Laser Technol..

[23] Kumar P.P., Bharat A.R., Sai B.S., Sarath R.P., Akhil P., Reddy G.P.K., Kondaiah V., Sunil B.R. (2019). Role of microstructure and secondary phase on corrosion behavior of heat treated AZ series magnesium alloys. Mater. Today: Proc..

[24] Sung Y.M., Lee Y.J., Park K.S. (2006). Kinetic analysis for formation of Cd1-x Zn x Se solid-solution nanocrystals. J. Am. Chem. Soc..

[25] Yeh J.W., Chang S.Y., Hong Y.D., Chen S.K., Lin S.J. (2007). Anomalous decrease in X-ray diffraction intensities of Cu–Ni–Al–Co–Cr–Fe–Si alloy systems with multi-principal elements. Mater. Chem. Phys..

[26] Shang C., Axinte E., Sun J., Li X., Li P., Du J., Wang Y. (2017). CoCrFeNi(W1−xMox) high-entropy alloy coatings with excellent mechanical properties and corrosion resistance prepared by mechanical alloying and hot pressing sintering. Mater. Des..

[27] Regev M., Rosen A., Bamberger M. (2001). Qualitative model for creep of AZ91D magnesium alloy. Metall. Mater. Trans..

[28] Maeshima T., Oh-Ishi K. (2019). Solute clustering and supersaturated solid solution of AlSi10Mg alloy fabricated by selective laser melting. Heliyon.

[29] Yang Y., Lu C., Shen L., Zhao Z., Peng S., Shuai C. (2021). In-situ deposition of apatite layer to protect Mg-based composite fabricated via laser additive manufacturing. J. Magnes. Alloy..

[30] Li J., Qiu Y., Yang J., Sheng Y., Yi Y., Zeng X., Chen L., Yin F., Su J., Zhang T. (2021). Effect of grain refinement induced by wire and arc additive manufacture (WAAM) on the corrosion behaviors of AZ31 magnesium alloy in NaCl solution. J. Magnes. Alloy..

[31] Xia Y.H., Zhang B.P., Lu C.X., Geng L. (2013). Improving the corrosion resistance of Mg-4.0Zn-0.2Ca alloy by micro-arc oxidation. Mater. Sci. Eng..

[32] Tamilselvi S., Rajendran N. (2007). In vitro corrosion behaviour of Ti-5Al-2Nb-1Ta alloy in Hanks solution. Mater. Corros..

[33] Klein F., Bach W., Jöns N., McCollom T., Moskowitz B., Berquó T. (2009). Iron partitioning and hydrogen generation during serpentinization of abyssal peridotites from 15° N on the Mid-Atlantic Ridge. Geochim. Cosmochim. Acta.

[34] Yang Y., He C., Dianyu E., Yang W., Qi F., Xie D., Shuai C. (2020). Mg bone implant: Features, developments and perspectives. Mater. Des..

[35] Atrens A., Johnston S., Shi Z., Dargusch M. (2018). Viewpoint-Understanding Mg corrosion in the body for biodegradable medical implants. Scr. Mater..

[36] Qian G., Zhang L., Wang G., Zhao Z., Peng S., Shuai C. (2021). 3D Printed Zn-doped Mesoporous Silica-incorporated Poly-L-lactic Acid Scaffolds for Bone Repair. Int. J. Bioprinting.

[37] Song M.-S., Zeng R.-C., Ding Y.-F., Li R., Easton M., Cole I., Birbilis N., Chen X.-B. (2019). Recent advances in biodegradation controls over Mg alloys for bone fracture management: A review. J. Mater. Sci. Technol..

[38] Zhang Y., Li J., Li J. (2017). Effects of calcium addition on phase characteristics and corrosion behaviors of Mg-2Zn-0.2 Mn-xCa in simulated body fluid. J. Alloy. Compd..

[39] Yan K., Bai J., Liu H., Jin Z.-Y. (2017). The precipitation behavior of MgZn2 and Mg4Zn7 phase in Mg-6Zn (wt.%) alloy during equal-channel angular pressing. J. Magnes. Alloy..

[40] Xiao C., Wang L., Ren Y., Sun S., Zhang E., Yan C., Qin G. (2018). Indirectly extruded biodegradable Zn-0.05 wt% Mg alloy with improved strength and ductility: In vitro and in vivo studies. J. Mater. Sci. Technol..

[41] Qi F., Zeng Z., Yao J., Cai W., Zhao Z., Peng S., Shuai C. (2021). Constructing core-shell structured BaTiO3@ carbon boosts piezoelectric activity and cell response of polymer scaffolds. Mater. Sci. Eng..

[42] Gao C., Yao M., Peng S., Tan W., Shuai C. (2021). Pre-oxidation induced in situ interface strengthening in biodegradable Zn/nano-SiC composites prepared by selective laser melting. J. Adv. Res..

